# Ergothioneine Thione Spontaneously Binds to and Detaches from the Membrane Interphase

**DOI:** 10.3390/membranes15110328

**Published:** 2025-10-29

**Authors:** José Villalaín

**Affiliations:** Institute of Research, Development, and Innovation in Healthcare Biotechnology (IDiBE), Universitas “Miguel Hernández”, E-03202 Alicante, Spain; jvillalain@umh.es

**Keywords:** ergothioneine, antioxidant, molecular dynamics, membrane

## Abstract

Ergothioneine is a potent non-toxic and very stable antioxidant which is synthesized by fungi, algae, and bacteria but not animals or higher plants. Ergothioneine has been widely used in cosmetics; dietary supplements; and medicine to treat diabetes, cancer, as well as cardiovascular, neurodegenerative, and liver diseases. Ergothioneine presents two tautomeric forms: thione, the majoritarian and more stable form (ERGO), and thiol (ERGT). Ergothioneine cannot cross cell membranes, and human cells rely on a specific transporter, OCTN1, to transport ingested ERGO to different parts of the body. Ergothioneine is very hydrophilic, and it is supposed to act at the water level but not at the membrane one. In this work, I studied the interaction of ERGO and ERGT with a complex biomembrane using molecular dynamics (MD). MD suggests that ERGO, but not ERGT, inserts spontaneously into the membrane interphase and can move from the membrane interphase to the water phase and vice versa, and no oligomerization was observed. Furthermore, ERGO, when inserted in the membrane, does not alter the hydrocarbon chain order. Therefore, ERGO (the thione form of ergothioneine), but not ERGT (the thiol form), might act at both the water and membrane interphase levels.

## 1. Introduction

**Figure 1:**
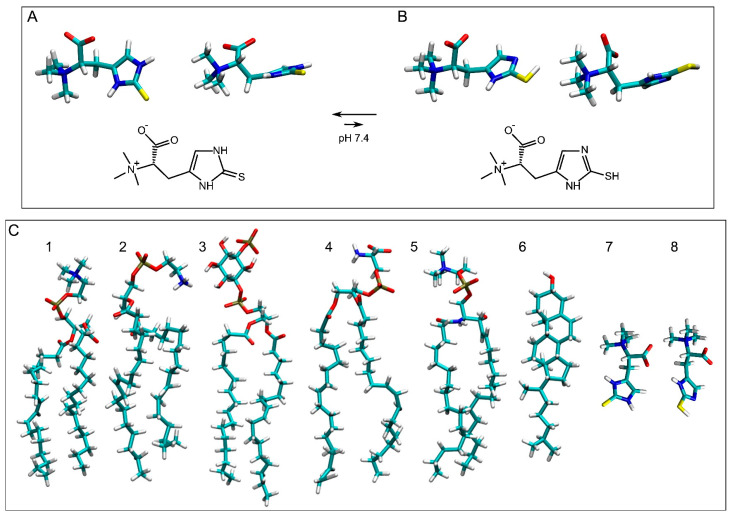
Chemical and molecular structures of (**A**) ERGO and (**B**) ERGT. The molecular structures of the molecules studied in this work are shown in (**C**) ((1) POPC, (2) POPE, (3) PI-3P, (4) POPS, (5) PSM, (6) CHOL, (7) ERGO, and (8) ERGT) to compare molecular sizes. The molecules are shown in licorize form.

Ergothioneine (Figure 1A), an essential physiological molecule discovered more than one hundred years ago, is synthesized by fungi, algae, and bacteria but not animals or higher plants [1,2,3,4,5,6]. Ergothioneine, among other properties, is a potent antioxidant and has been widely used in cosmetics, dietary supplements, and medicine [1,5,7,8,9,10,11,12,13,14]. Ergothioneine is non-toxic and very stable and has been used to treat diabetes, cancer, and cardiovascular, neurodegenerative, and liver diseases [5,11,14,15,16,17,18,19,20,21,22,23,24,25,26,27]. Ergothioneine is safe, and different international administrations (European Commission, USA, China) have approved its use as a food ingredient [9,28]. Because of that, many different beverages and foods in the market include ergothioneine in them [29].

Oxidative stress leads to neurodegeneration and ageing, both of which pose a major challenge to human health [30]. Antioxidants aim to stop the loop between oxidative stress, inflammation, protein misfolding, and lipid peroxidation that together cause a number of diseases [31]. Ergothioneine is a molecule derived from histidine (2-mercaptohistidine trimethylbetaine) and presents two tautomeric forms: thione, the major and more stable form (ERGO), and thiol (ERGT) (Figure 1A and Figure 1B, respectively) [32]. This occurs because under physiological conditions, the thiocarbonyl group of ERGO is more stable than the sulfhydryl group of ERGT [28,32,33,34]. Moreover, ERGO has a greater resistance to autooxidation than ERGT, and additionally, ERGO has greater thermal and chemical stability than other biological thiols such as glutathione [32,34,35]. ERGO and ERGT, being low-molecular-mass molecules and containing polar groups, are highly soluble in water [32]. Ergothioneine is noticeably different from glutathione due to its lower redox potential, tautomerization, and potent antioxidant properties [9]. Significantly, ergothioneine can easily chelate divalent metal ions to form inactive ergothioneine–metal complexes and is a potent scavenger of reactive oxygen and nitrogen species, which are known to damage lipids, proteins, and nucleic acids, altering their functions [11,36].

Ergothioneine is a recognized antioxidant, even at low concentrations, but human cells cannot synthesize it [34,36]. The highest dietary sources of ergothioneine are edible mushrooms, and many tissues are capable of accumulating ergothioneine at relatively high concentrations [28,34,36]. Ergothioneine cannot cross cell membranes, and human cells rely on a specific transporter, OCTN1 (now known as SLC22A4 [37]), to transport dietary ingested ergothioneine to different parts of the body, including liver, spleen, kidney, heart, eyes, and brain, which would suggest that ergothioneine is a fundamental nutrient [5,9,28,38,39,40]. OCTN1 increases the initial cellular uptake of ergothioneine, which cannot pass through membranes due to its hydrophilic nature, by several hundred times [41]. Moreover, ergothioneine is taken up and accumulated in mitochondria [20]. It has also been described that the ergothioneine concentration in the body differs between different tissues and tends to be increased in areas of inflammation or injury [41,42,43]. If that were true, ergothioneine should be considered an adaptative antioxidant [41]. Ergothioneine, apart from its antioxidant properties, has been reported to be an anti-inflammatory, anti-apoptotic, anti-ageing, neuroprotective, and chelating agent [5,9,10,11,14,15,16,17,18,19,20,21,22,23,24,25,27,28,36,44,45,46]. Very recently, it has been described that ergothioneine can activate cytosolic glycerol-3-phosphate dehydrogenase, which is directly related to different ageing processes [45]. Moreover, ergothioneine is metabolized and excreted slowly [47]. Interestingly, one molecule of ergothioneine can reduce two molecules of hydrogen peroxide, twice as much as ascorbic acid [48]. Furthermore, the ergothioneine oxidation product, hercynine, is also a good antioxidant molecule [48]. Ergothioneine is therefore a promising molecule for the treatment of many different diseases [5,28,36]. Thus, ergothioneine is a stable, safe, and efficient antioxidant molecule with significant potential for human and animal health. From these data, is clear that ergothioneine is a bioactive vitamin-like molecule that is important for human and animal health.

It is known that, in general, lipophilic antioxidants, such as vitamin E, astaxanthin, or coenzyme Q, protect lipid peroxidation, whereas hydrophilic antioxidants, such as vitamin C or glutathione, protect proteins [27,28,49,50]. Recent data suggest that ergothioneine is coupled with the glutathione and vitamin C redox cycles [13]. ERGO is hydrophilic (Figure 1A) and has a consensus octanol–water partition coefficient log P value of −1.56 (http://www.swissadme.ch/, accessed on 18 August 2025). ERGT is also hydrophilic (Figure 1B) and has a slightly higher consensus log P value, −1.60 (i.e., it is a bit more hydrophilic than ERGO). Due to its hydrophilicity, ergothioneine has been proposed to act at the water level but not at the membrane level. In this work, I studied the possible interaction of both ERGO and ERGT with a complex biomembrane by molecular dynamics (MD). MD is appropriate to attain suitable information on the structure, dynamics, position, and interactions of bioactive molecules inside membranes [51,52,53,54,55] (Table 1). In this work, I did not study the interaction between ERGO/ERGT and OCTN1 but the interaction between those molecules and a complex membrane. I show for the first time that ERGO, but not ERGT, is not only able to bind to the membrane surface but is also able to insert spontaneously into the membrane interphase, i.e., the space between the phospholipid phosphate atoms and the cholesterol oxygen atom, always in the monomeric state. Furthermore, ERGO can cross to the aqueous phase from the membrane interface and vice versa. Therefore, the antioxidant properties of ERGO are not circumscribed only to the water level but also to the membrane surface. In this way, ERGO can act as a bridge between antioxidants in a solution and antioxidants located in the membrane.

## 2. Materials and Methods

Unrestrained all-atom MD was performed using NAMD (Nanoscale Molecular Dynamics) 3.0b2 [56], as well as the CHARMM36 protein and lipid force fields [57,58,59]. All MD parameters have been previously described [53,54,55]. The systems were minimized for 150,000 steps to eliminate bad atomic contacts and then equilibrized for 10 ns. The production trajectories for each of the systems were run for a total of 800 ns (Table 1).

**Figure 2:**
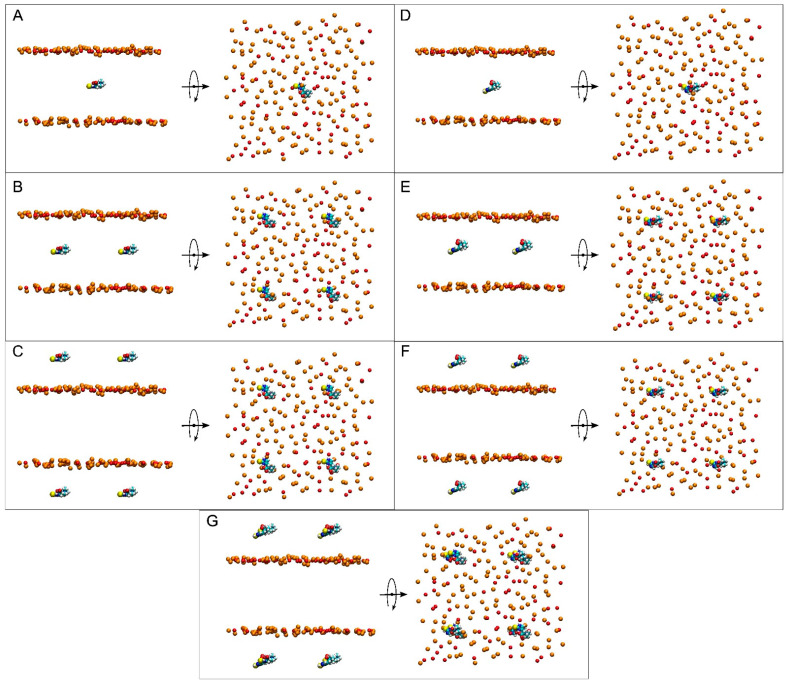
Lateral and apical views of the initial, t = 0 ns, dispositions of (**A**) system 1, (**B**) system 2, (**C**) system 3, (**D**) system 4, (**E**) system 5, (**F**) system 6, and (**G**) system 7. ERGO and ERGOT molecules are presented in a VDW representation, whereas the phosphate atoms of the phospholipids, defining the upper and lower boundaries of the membrane, and the oxygen atoms of CHOL are represented in VDW and orange and red colours, respectively. The water and lipid molecules and the chloride and sodium ions have been removed for clarity.

The multicomponent membrane systems were obtained using Charmm-Gui (http://www.charmm-gui.org, accessed on 19 May 2025 [60]). Systems contained NaCl at physiological conditions, i.e., a concentration of 150 mM and an excess of water [61] in a neutral environment (Table 1) [62,63,64]. Membranes were like the plasma membrane (Table 1) and contained 56 molecules of 1-palmitoyl-2-oleoyl-sn-glycero-3-phosphocholine (POPC), 34 molecules of 1-palmitoyl-2-oleoyl-sn-glycero-3-phosphoethanolamine (POPE), 14 molecules of 1-palmitoyl-2-oleoyl-sn-glycero-3-phosphoserine (POPS), 12 molecules of 1-palmitoyl-2-oleoyl-sn-glycero-3-phosphoinositol-3-phosphorous (PI-3P), 24 molecules of N-stearoyl-D-erythro-sphingosylphosphorylcholine (PSM), and 60 molecules of cholesterol (CHOL) (Table 1) [65,66]. For ERGO, three systems were studied: a membrane system containing one ERGO molecule, system 1 (Figure 2A); a membrane system containing four ERGO molecules, system 2 (Figure 2B); and a membrane system containing eight ERGO molecules, system 3 (Figure 2C). Similarly, for ERGT, three systems were studied: a membrane system containing one ERGT molecule, system 4 (Figure 2D); a membrane system containing four ERGT molecules, system 5 (Figure 2E); and a membrane system containing eight ERGT molecules, system 6 (Figure 2F). In addition, system 7 contained a membrane plus four ERGO and four ERGT molecules (Figure 2G). The lipid chemical structures are shown in Figure 1C. Membrane fluidity was augmented using one oleoyl hydrocarbon chain in the phospholipids [53,54]. PSM, apart from the sphingosyl chain, contained a palmitoyl one. The molecular structure of ERGO was obtained from PubChem (https://pubchem.ncbi.nlm.nih.gov/compound/5351619, accessed on 29 April 2025) and was revised and minimized using Discovery Studio 4.0 (Accelrys Inc., San Diego, CA, USA). ERGT was obtained, revised, and minimized from ERGO, also using Discovery Studio 4.0 (Accelrys Inc., San Diego, USA). The CHARMM General Force Field stream files of both ERGO and ERGT were attained with Charmm-Gui (http://www.charmm-gui.org, accessed on 19 May 2025 [60]).

VMD (Visual Molecular Dynamics, Theoretical and Computational Biophysics Group, University of Illinois at Urbana-Champaign) and VMD plugins were used for analysis [67,68,69,70,71,72,73,74,75].

## 3. Results and Discussion

Seven different membrane systems were studied: three systems containing ERGO, three systems containing ERGT, and one system containing both ERGO and ERGT. Systems 1 and 4 contained a complex membrane and only one ERGO and ERGT molecule, respectively (Figure 2A,D); systems 2 and 5 contained a complex membrane and four ERGO and ERGT molecules, respectively (Figure 2B,E); systems 3 and 6 contained a complex membrane and eight ERGO and ERGT molecules, respectively (Figure 2C,F); and system 7 contained a complex membrane and four ERGO and four ERGT molecules (Figure 2G). Both ERGO/ERGT molecules in systems 1, 2, 4, and 5 at time zero were positioned at the membrane centre, while the ERGO/ERGT molecules in systems 3, 6, and 7 were positioned outside the membrane, centred in each of the water layers (Figure 2) [55,76,77]. All these systems were very diluted, since the membrane was composed of 200 lipids, with 100 in each layer. I studied the bilayer thickness and lipid molecular areas to evaluate the equilibration of the systems during the MD simulations [73,78,79]. After ~100−120 ns, the thickness was similar for all systems, being undistinguishable for the last 30 ns, i.e., between 46 and 47 Å for the thickness corresponding to the phospholipid phosphates (Figure S1 and Table S1). Data obtained are akin to those already described [80]. The mean areas for the last 30 ns of MD and for all systems for POPC, POPE, POPS, and PI-3P were among 49 and 53 Å^2^; for PSM, they fluctuated between 47 and 49 Å^2^; and for CHOL, they fluctuated between 27 and 28 Å^2^ (Figure S2 and Table S1). They were similar to those described earlier [71,80,81]. Hence, all systems were stabilized and equilibrated after approximately ~100−120 ns of MD.

**Figure 3:**
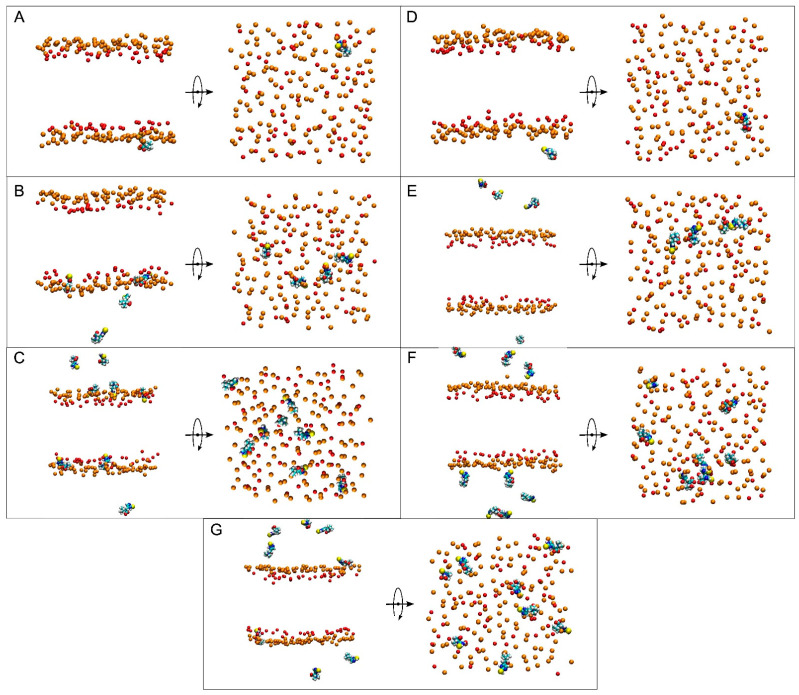
Lateral and apical views of the final, t = 450 ns, dispositions of (**A**) system 1, (**B**) system 2, (**C**) system 3, (**D**) system 4, (**E**) system 5, (**F**) system 6, and (**G**) system 7. ERGO and ERGOT molecules are presented in a VDW representation, whereas the phosphate atoms of the phospholipids—defining the upper and lower boundaries of the membrane—and the oxygen atoms of CHOL are represented in VDW and orange and red colours, respectively. The water and lipid molecules and the chloride and sodium ions have been removed for clarity.

**Figure 4:**
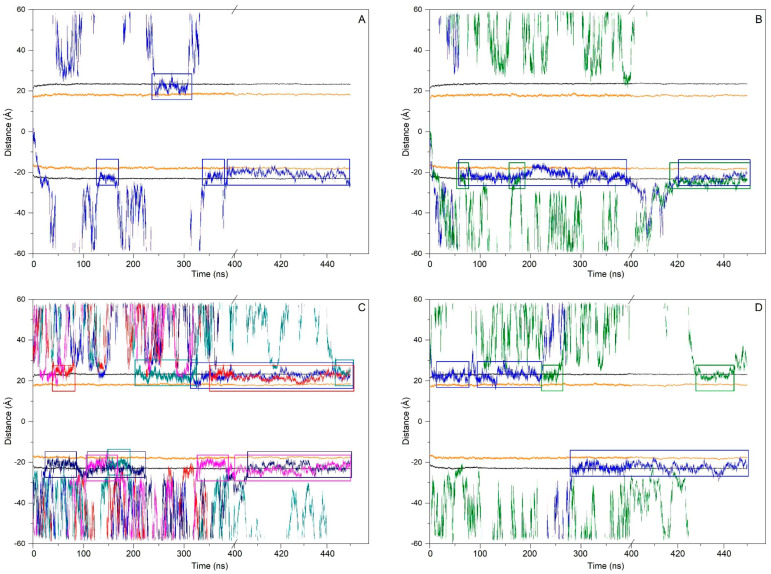
Time variation in the whole-molecule ERGO z-axis COM distance in relation to (**A**) system 1, (**B**) system 2, (**C**) system 3, and (**D**) system 7. Only ERGO molecules bound to the membrane for the last 30 ns of MD are shown. Each ERGO molecule is represented by a different colour. Coloured boxes mark the ERGO molecules that are bound to the membrane interphase along the whole MD. The phosphate atoms of the phospholipids and the oxygen atom of cholesterol are shown in black and orange colours, respectively.

**Figure 5:**
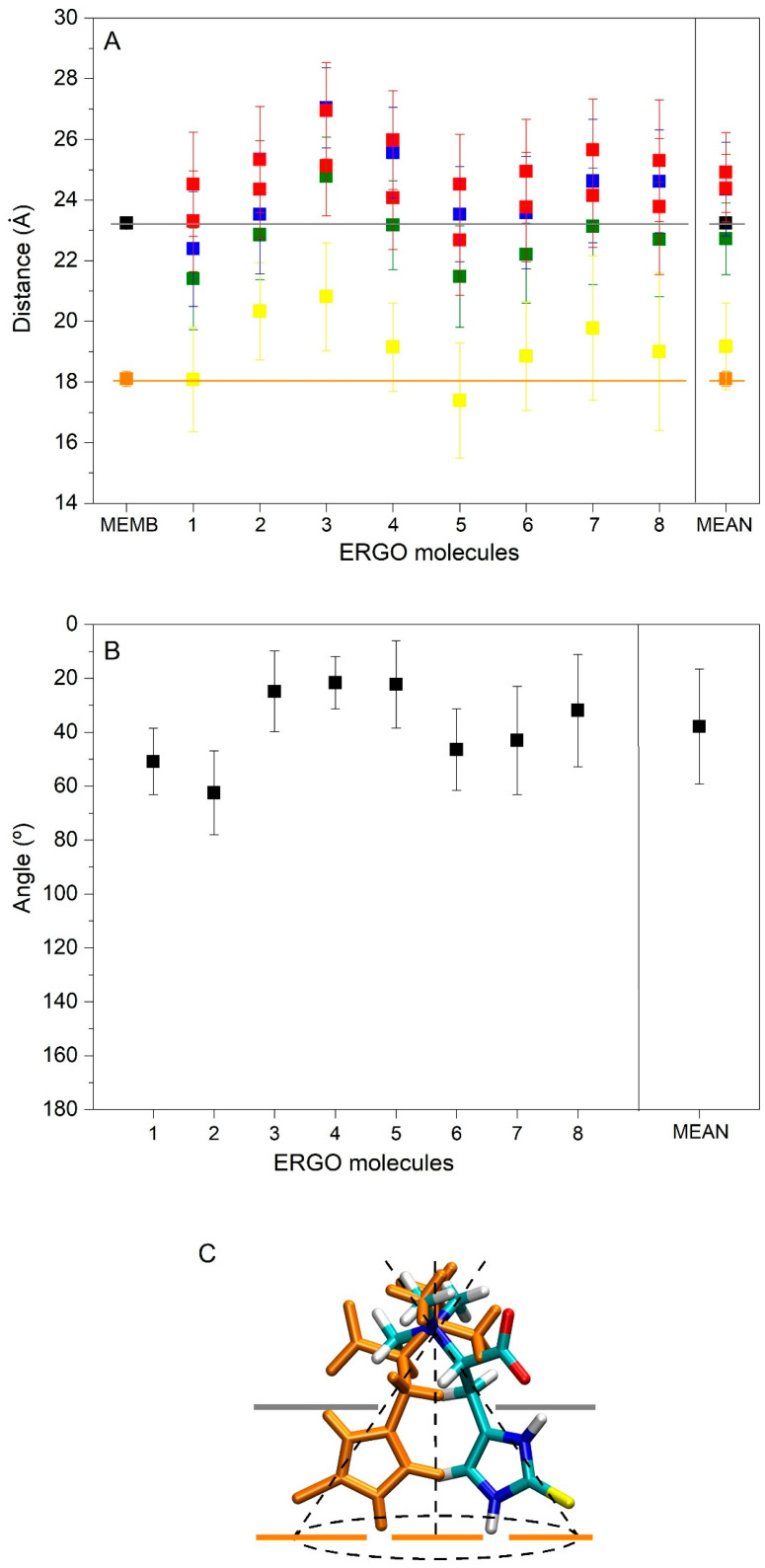
(**A**) Average z-axis COM distance for the last 30 ns of MD simulation in relation to the trimethylammonium nitrogen (■), oxygen atoms (■), and sulphur (■) atoms of ERGO, the whole ERGO molecule (■), as well as the phosphate atoms of the phospholipids (■) and the oxygen atom of cholesterol (■). (**B**) Average angle of the trimethylammonium nitrogen and sulphur atoms of ERGO molecules with respect to the perpendicular membrane for the last 30 ns of MD simulation (■). Molecule 1 belongs to system 1, molecules 2 and 3 to system 2, molecules 4−7 to system 3, and molecule 8 to system 7. (**C**) Representation of the ERGO molecule in the membrane and the average angle that it forms with it. Grey and orange lines in (A) and (C) represent the average COM distance of the phosphate atoms of the phospholipids and the oxygen atom of cholesterol, respectively.

At the beginning, system 1 had one ERGO molecule at the membrane centre (Figure 2A). At the end of the MD, the ERGO molecule moved to a site next to the bilayer interphase (Figure 3A). Its behaviour can be studied knowing its z-axis centre-of-mass (z-COM, z direction to the bilayer plane, with the centre of the membrane as a reference) (Figure 4A and Figure S3A). As said above, the ERGO molecule at 0 ns was placed in the middle of the membrane; it rapidly moved to the water phase (Figure 4A). With time, it moved into and out of the membrane interphase—sometimes the interphase of one monolayer, sometimes the other (see boxes, Figure 4A). As observed in Figure 4A, the molecule was able to move from the water phase to the membrane interphase and from the membrane interphase to the water phase. Significantly, at no time did the ERGO molecule cross the middle part of the membrane from one monolayer to the other (Figure 4A). The average z-COM distances for the last 30 ns of MD simulation of the trimethylammonium nitrogen and oxygen and sulphur atoms of ERGO were 22.4 ± 1.9 Å, 23.3 ± 1.6 Å, 24.5 ± 1.7 Å, and 18.1 ± 1.7 Å, respectively (Figure 5A, molecule 1). The average location of the sulphur atom is on par with the oxygen atoms of CHOL, whereas the trimethylammonium nitrogen and oxygen atoms of ERGO are located at the phosphate atom level.

System 2 had four ERGO molecules at the membrane centre (Figure 2B). The ERGO molecules were separated by 40 Å in the xy-plane. During the MD, the ERGO molecules moved to and through the water phase, as well as the membrane interphase, so that at the end of the MD, two ERGO molecules were bound to the membrane interphase and two were located at the water phase (Figure 3B). Their behaviour throughout the MD can be studied observing the z-COM data of the four ERGO molecules (Figure 4B and Figure S3B). Similarly to the ERGO molecule in system 1, the ERGO molecules moved in and out of the membrane interphase—sometimes into the interphase of one monolayer, sometimes into the other (see boxes in Figure 4B, as well as Figure S3B). As observed in Figure 4B, the molecules were able to move from the water phase to the membrane interphase and from the membrane interphase to the water phase. At no time did the ERGO molecules cross the middle part of the membrane from one monolayer to the other (Figure S3B). The average z-COM distances for the last 30 ns of the MD simulation of the trimethylammonium nitrogen and oxygen and sulphur atoms of ERGO were 23.5 ± 1.9 Å and 27.0 ± 1.3 Å, 25.3 ± 1.7 Å, and 26.9 ± 1.6 Å; 24.3 ± 1.6 Å and 25.1 ± 1.6 Å; and 20.3 ± 1.6 Å and 20.8 ± 1.8 Å, respectively (Figure 5A, molecules 2 and 3). The average location of the sulphur atoms is between the oxygen atoms of CHOL and the phosphate atoms of the phospholipids, whereas the trimethylammonium nitrogen and oxygen atoms are located above the phosphate atom level. At no point was oligomerization between ERGO molecules observed.

System 3 had eight ERGO molecules: four molecules in the middle of each water layer (Figure 2C). The ERGO molecules were separated by 40 Å in the xy-plane and 65 Å in the z-plane. During the MD, the ERGO molecules moved to and through the water phase, as well as the membrane interphase, so that at the end of the MD, five ERGO molecules were bound to the membrane interphase, and three were located at the water phase (Figure 3C). Their behaviour throughout the MD is shown in Figure 4C and Figure S3C. Similarly to the ERGO molecules in systems 1 and 2, the ERGO molecules moved into and out of the membrane interphase—sometimes into the interphase of one monolayer, sometimes into the other (see boxes in Figure 4C, as well as Figure S3C). As observed in Figure S3C, the molecules were able to move from the water phase to the membrane interphase and from the membrane interphase to the water phase. At no time did the ERGO molecules cross the middle part of the membrane from one monolayer to the other (Figure S3C). The average z-COM distances for the last 30 ns of MD simulation for the trimethylammonium nitrogen atoms were 25.5 ± 1.5, 23.5 ± 1.6, 23.6 ± 1.8 Å, and 24.6 ± 2.0 Å; for the oxygen atoms, they were 25.9 ± 1.6, 24.5 ± 1.6, 23.7 ± 1.8 Å and 24.1 ± 1.7 Å, and 24.1 ± 1.7, 22.7 ± 1.8, 24.9 ± 1.7 Å and 25.6 ± 1.7 Å; and for the sulphur atoms, they were 19.1 ± 1.4 Å, 17.4 ± 1.9, 18.8 ± 1.8, and 19.7 ± 2.4 Å (Figure 5A, molecules 4 to 7). The average location of the sulphur atoms lies slightly above the oxygen atoms of CHOL, whereas the trimethylammonium nitrogen and oxygen atoms are located slightly above the phosphate atom level. At no point was oligomerization between ERGO molecules observed.

At the beginning, system 4 had one ERGT molecule at the membrane centre (Figure 2D). At the end of the MD, the ERGT molecule had moved to the water phase (Figure 3D). Its z-COM behaviour is shown in Figure S3D. Very quickly, the ERGT molecule, situated in the centre of the membrane at t = 0 ns, moved to the aqueous phase, never returning to the membrane—neither the interphase or the interior. System 5 had four ERGT molecules at the membrane centre (Figure 2D). The ERGT molecules were separated by 40 Å in the xy-plane. At the end of the MD, the ERGT molecules had moved to the water phase (Figure 3D). Their z-COM behaviour is shown in Figure S3E. Very quickly, the ERGT molecules, situated in the centre of the membrane at t = 0 ns, moved to the aqueous phase, never returning to the membrane—neither the interphase or the interior. System 6 had eight ERGT molecules: four molecules in the middle of each water layer (Figure 2E). The ERGT molecules were separated by 40 Å in the xy-plane and 65 Å in the z-plane. At the end of the MD, all the ERGT molecules remained in the water phase (Figure 3E). Their z-COM behaviour is shown in Figure S3F. Very quickly, the ERGT molecules, situated in the centre of the membrane at t = 0 ns, moved to the aqueous phase, never returning to the membrane—neither the interphase or the interior. On some occasions, some of the ERGT molecules stayed near the phosphate atoms of the phospholipids, but they moved again to the water phase relatively quickly (Figure S3F). At no point was oligomerization between ERGT molecules observed.

At the beginning, system 7 had four ERGO and four ERGT molecules, with two of each in the middle of each water layer (Figure 2G). The ERGO molecules were separated by 40 Å in the xy-plane and 65 Å in the z-plane. During the MD, the ERGO and ERGT molecules moved to and through the water phase, as well as the membrane interphase; at the end of the MD, only two ERGO molecules were bound to the membrane interphase, while the other six ERGO/ERGT molecules were located in the water phases (Figure 3G). Their behaviour throughout the MD is shown in Figure S3G. Along the MD, all four ERGO molecules were sometimes absorbed into the membrane interphase, moving into and out of it, sometimes into the interphase of one monolayer, and sometimes into the other. At no time did the ERGO molecules cross the middle part of the membrane from one monolayer to the other (Figure S3G). Significantly, no ERGT molecules were inserted into the membrane surface along the MD, remaining in the water phase all the time. Only one ERGO molecule was bound for more than 30 ns at the end of the MD (Figure 4D). The average z-COM distances for this molecule and for the last 30 ns of the MD simulation of the trimethylammonium nitrogen and oxygen and sulphur atoms of this ERGO molecule were 24.6 ± 1.7 Å, 25.3 ± 2.0 Å, 23.8 ± 2.2 Å, and 19.0 ± 2.6 Å, respectively (Figure 5A, molecule 8). The average location of the sulphur atoms lies slightly above the oxygen atoms of CHOL, whereas the trimethylammonium nitrogen and oxygen atoms are located slightly above the phosphate atom level. At no point was oligomerization between the ERGO and ERGT molecules observed.

Summarizing the results obtained for all the systems studied here, ERGO molecules spontaneously insert into the membrane interphase; they can move from it to the water phase and vice versa, and they do not show oligomerization. However, and in contrast to ERGO, ERGT molecules do not insert into the membrane interphase at any time. The average z-COM distances for the ERGO molecules in the membrane interphase and for the last 30 ns of MD simulation were 24.3 ± 1.5 Å for the trimethylammonium nitrogen, 24.9 ± 1.3 Å and 24.4 ± 1.1 Å for the oxygen atoms, and 19.2 ± 1.4 Å for the sulphur atom (Figure 5A). The average location of the sulphur atoms lies slightly above the oxygen atoms of CHOL, whereas the trimethylammonium nitrogen and oxygen atoms are located slightly above the phosphate atom level.

As noted above, ERGO spontaneously inserts into the membrane interphase and always remains in the monomer state. Importantly, the mean location of the sulphur atoms lies slightly above the oxygen atoms of CHOL, whereas the trimethylammonium nitrogen and oxygen atoms are located slightly above the phosphate atom level. I determined the average angles formed by the trimethylammonium nitrogen and the sulphur atom of ERGO molecules at the membrane interphase with respect to the surface of the membrane (Figure 5B). As observed in the figure, the angles vary between about 20° and 60°, with the average being 37° ± 21° (Figure 5B). The approximate distribution of the molecule at the membrane interphase can be seen in Figure 5C. From the data presented in Figure 5A,B, it is clear that the ERGO molecule does not extend beyond the position of the cholesterol oxygens. Because of this, ERGO does not modify the hydrocarbon chain fluidity as measured by the phospholipid S_CD_ order parameter (results not shown). The lipid mass density profiles of all the systems studied in this work are shown in Figure S4. As expected, and since all the systems were very diluted, the lipid density profiles were symmetrical for the two monolayers, representing similar conduct of the lipids. In the figure, it is possible to observe the lipid mass density profiles of ERGO and ERGT with respect to the lipids. ERGO tends to be located between the phosphate atoms of the phospholipids and the oxygen atoms of CHOL, whereas ERGT might be near the membrane surface, but at no time do they not move beyond the phospholipid phosphate atoms. To ascertain if any lipid type is increased or reduced close to ERGO, I assessed the number and type of lipid molecules within a distance of 5 Å from ERGO and related them to the bulk numbers (Figure S5). The proportion of POPC was higher than the bulk proportions (42% near ERGO vs. bulk 28%), whereas the proportions of CHOL and PSM were lower (30% vs. 20% and 12% vs. 7%). The proportions of POPE, POPS, and PI-P3 near ERGO were similar to the bulk percentages. In general, it can be said that ERGO avoids PSM and CHOL and prefers the POPC (Figure S5). The average number of hydrogen bonds between ERGO and the lipids are shown in Figure S6. The values are relatively low, except for POPE (0.8 ± 0.4 for POPE, whereas it was 0.3 ± 0.3 for CHOL, 0.2 ± 0.2 for POPC, 0.1 ± 0.3 for PSM, and 0 for both POPS and PI-2P).

## 4. Conclusions

**Figure 6:**
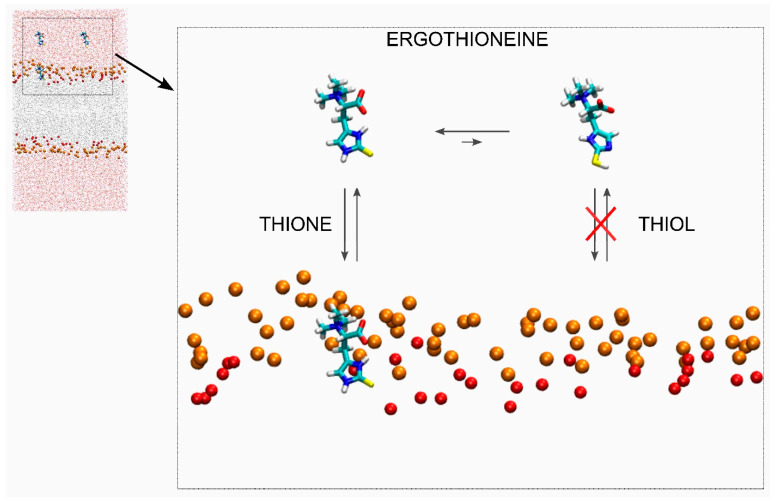
Schematic representation of the movement and location of ERGO and ERGT in the presence of a membrane. The ERGO and ERGT molecules are presented in liquid form. The phosphate atoms of the phospholipids (orange, VDW size reduced by half), defining the upper and lower boundaries of the membrane, and the oxygen atoms of cholesterol (red, VDW size reduced by half). For clarity, lipid atoms, water, and chloride and sodium ions have been removed.

Ergothioneine is a potent antioxidant that is synthesized only by fungi, algae, and bacteria. In medicine, ergothioneine has been used to treat diabetes, as well as cancer and cardiovascular, neurodegenerative, and liver diseases. Ergothioneine presents two tautomeric forms: thione, the majoritarian and more stable form (ERGO), and thiol (ERGT). Ergothioneine cannot cross cell membranes, and human cells rely on a specific transporter, OCTN1, to transport ingested ergothioneine to different parts of the body. Supposedly, ergothioneine, being a very hydrophilic molecule, acts at the water level but not at the membrane one. In this work, I studied the interaction of both ERGO and ERGT with a complex biomembrane by molecular dynamics. This computational model suggests that ERGO, but not ERGT, inserts spontaneously into the membrane interphase (Figure 6). Furthermore, ERGO can move from the membrane interphase to the water phase and vice versa, and no oligomerization is observed. The average location of the sulphur atoms of ERGO is slightly above the oxygen atoms of CHOL, whereas the trimethylammonium nitrogen and oxygen atoms are located slightly above the phosphate atom level. ERGO, when inserted in the membrane, forms an average angle of about 37°, does not alter the hydrocarbon chain order, and tends to be surrounded by POPC, but it forms hydrogen bonds with POPE. These results would imply that ERGO, but not ERGT, acts at both the water and membrane levels. In this way, ERGO can act as a bridge between antioxidants in a solution and antioxidants located in the membrane.

## Figures and Tables

**Table 1:** Membrane systems and number of components. The NaCl concentration was 0.15 M. The time to obtain the production trajectories for each of the systems is also indicated. The total number of lipid molecules was 200, with 100 per monolayer.

SYSTEM		1	2	3	4	5	6	7
ERGO/ERGT		1/0	-	-	-	-	-	-
ERGO/ERGT		-	4/0	-	-	-	-	-
ERGO/ERGT		-	-	8/0	-	-	-	-
ERGO/ERGT		-	-	-	0/1	-	-	-
ERGO/ERGT		-	-	-	-	0/4	-	-
ERGO/ERGT		-	-	-	-	-	0/8	-
ERGO/ERGT		-	-	-	-	-	-	4/4
MD time (ns)		450	450	450	450	450	450	450
POPC	28.8%	56	56	56	56	56	56	56
POPE	17.6%	34	34	34	34	34	34	34
PI-3P	5.6%	12	12	12	12	12	12	12
POPS	6.4%	14	14	14	14	14	14	14
PSM	12%	24	24	24	24	24	24	24
CHOL	29.6%	60	60	60	60	60	60	60
ATOMS		54,787	54,880	54,721	54,787	54,880	54,682	54,718
H_2_O		10,649	10,650	10,557	10,649	10,650	10,544	10,556
H_2_O/LIPID		53.2	53.2	52.8	53.2	53.2	52.7	52.8
Na^+^		80	80	80	80	80	80	80
Cl^−^		30	30	30	30	30	30	30
INITIAL DIMENSIONS x/y/z (Å)		78/78/118	78/78/118	78/78/118	78/78/118	78/78/118	78/78/118	78/78/118

## Data Availability

The original contributions presented in this study are included in the article and Supplementary Material. Further inquiries can be directed to the corresponding author.

## Supplementary Materials

The following supporting information can be downloaded at https://www.mdpi.com/article/10.3390/membranes15110328/s1: Figure S1: Time variation in membrane thickness for the whole simulation time and the corresponding histograms for the last 30 ns of MD simulation (the mean ± SD is also shown). Thickness corresponds to the phosphate atoms of the phospholipids for system 1 (grey), system 2 (red), system 3 (blue), system 4 (orange), system 5 (green), system 6 (wine red), and system 7 (violet); Figure S2: Time variation in lipid areas for the whole simulation time for (A) system 1, (B) system 2, (C) system 3, (D) system 4, (EE) system 5, (F) system 6, and (G) system 7. (H) Area average (± SD) for the last 30 ns of MD simulation. Colours correspond to POPC (dark grey), POPE (blue), POPS (red), PI-3P (green), PSM (wine red), and CHOL (orange); Figure S3: Time variation in the whole-molecule ERGO and ERGT z-axis COM distance in relation to (A) system 1, (B) system 2, (C) system 3, (D) system 4, (E) system 5, (F) system 6, and (G) system 7. ERGO and ERGT molecules are represented by different colours. The phosphate atoms of the phospholipids and the oxygen atom of cholesterol are shown in black and orange colours, respectively; Figure S4: Mass density profiles for the last 30 ns of the MD for (A) system 1, (B) system 2, (C) system 3, (D) system 5, (E) system 6, (F) system 7, and (G) system 7. The phosphate atoms of the phospholipids and the oxygen atoms of CHOL: POPC (**-**), POPE (**-**), POPS (**-**), PI-3P (**-**), PSM (**-**), and CHOL (**-**). The ERGO and ERGT molecules are shown in grey and red filled curves; Figure S5: Percentage of the number of lipid molecules in the membrane versus observed number of lipid molecules at a distance of 5 Å from the ERGO molecule in systems 1, 2, 3, and 7: POPC (-■-), POPE (-■-), POPS (-■-), PI-3P (-■-), PSM (-■-), and CHOL (-■-). The dotted line represents identical observed versus expected number of lipid molecules. The analysis was carried out for the last 30 ns of simulation; Figure S6: Mean number of hydrogen bonds between membrane lipids and ERGO (eight molecules from systems 1, 2, 3, and 7). The analysis was carried out for the last 30 ns of simulation, and uncertainties represent standard deviation; Table S1: Average molecular area (Å^2^) and membrane thickness (Å) for the last 40 ns of the simulation for all the lipids in the systems studied in this work (mean ± SD).

## Abbreviations

The following abbreviations are used in this manuscript:

ERGO	Ergothioneine (thione form)
ERGT	Ergothioneine (thiol form)
CHOL	Cholesterol
MD	Molecular dynamics
PI-3P	1-Palmitoyl-2-oleoyl-sn-glycero-3-phosphoinositol-3-phosphate
POPC	1-Palmitoyl-2-oleoyl-sn-glycero-3-phosphocholine
POPE	1-Palmitoyl-2-oleoyl-sn-glycero-3-phosphoethanolamine
POPS	1-Palmitoyl-2-oleoyl-sn-glycero-3-phospho-L-serine
PSM	N-Palmitoyl-D-erythro-sphingosylphosphorylcholine
z-COM	z-axis Centre-of-Mass (z direction normal to the bilayer plane)
