# Supplementary material for: Ergothioneine Thione Spontaneously Binds to and Detaches from the Membrane Interphase
Source: Membranes (Basel). 2025 Oct 29;15(11):328. doi: 10.3390/membranes15110328 (PMC12654265; doi:10.3390/membranes15110328)
Supplement: Supplementary file 1 [file membranes-15-00328-s001.zip › membranes-3905700-supplementary.pdf]

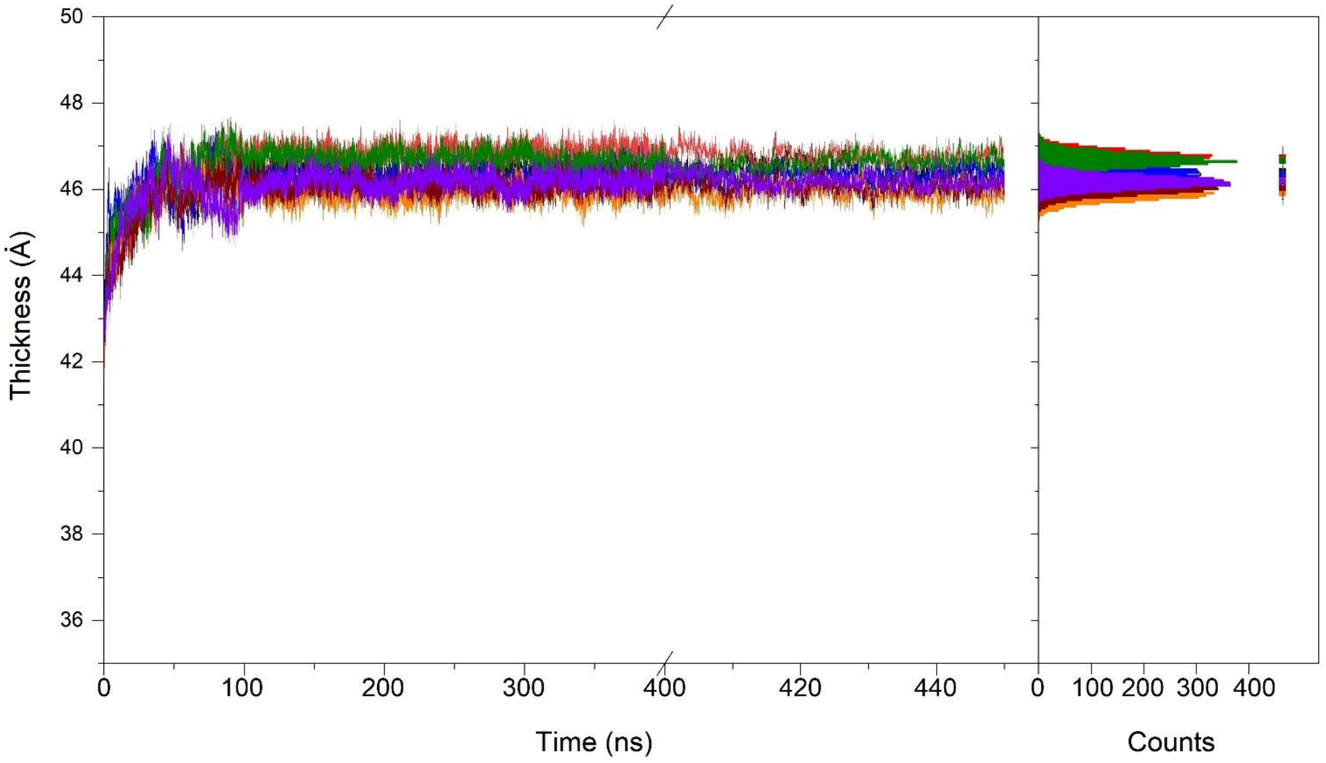

Figure S1. Time variation in membrane thickness for the whole simulation time and the corresponding histograms for the last 30 ns of MD simulation (the mean ± SD is also shown). Thickness corresponds to the phosphate atoms of the phospholipids for system 1 (grey), system 2 (red), system 3 (blue), system 4 (orange), system 5 (green), system 6 (wine red) and system 7 (violet).

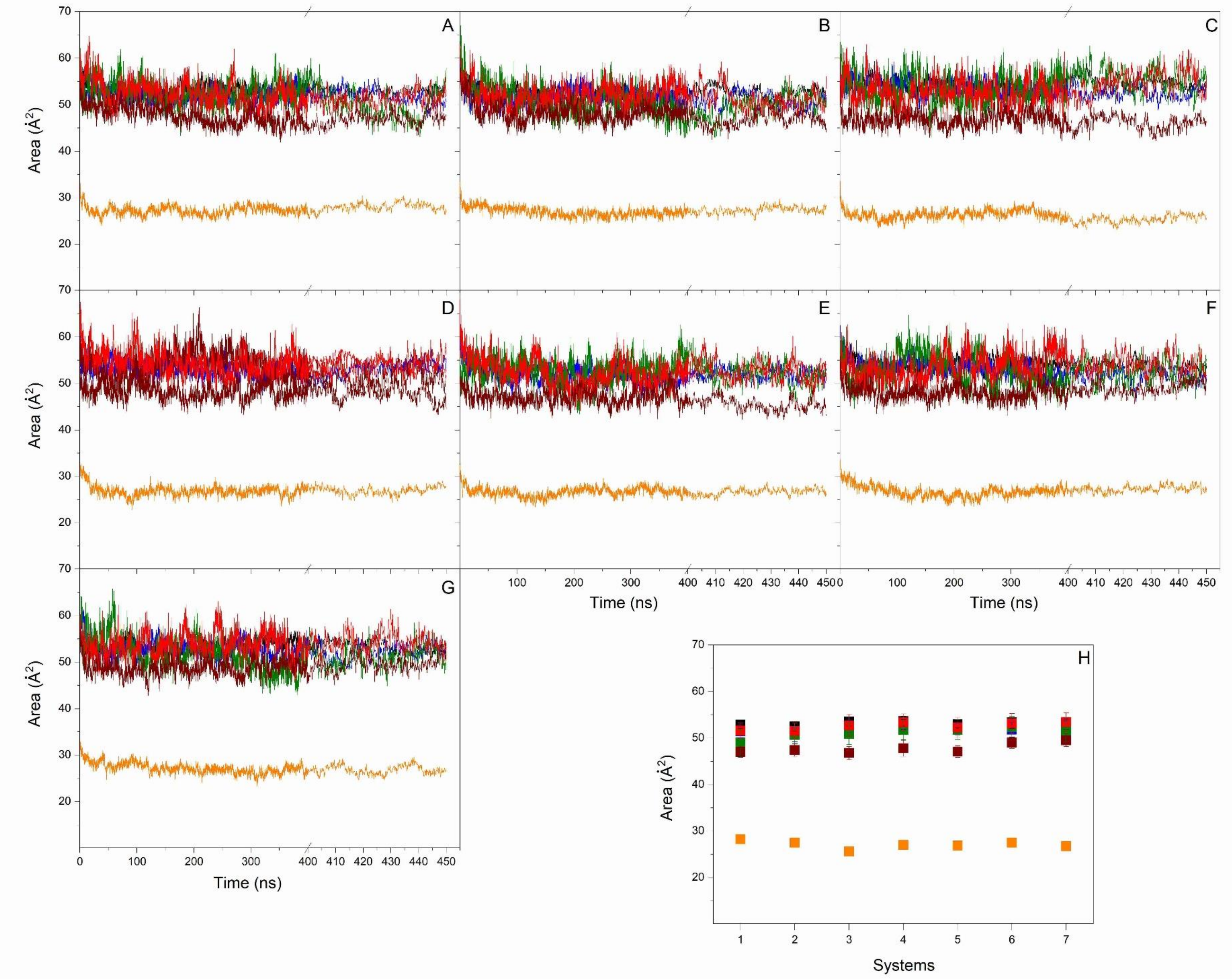
A
B
C
D
E
F
G
H
Area (Å^2)
Time (ns)
Systems

Figure S2. Time variation in lipid areas for the whole simulation time for (A) system 1, (B) system 2, (C) system 3, (D) system 4, (EE) system 5, (F) system 6, and (G) system 7. (H) Area average (± SD) for the last 30 ns of MD simulation. Colours correspond to POPC (dark grey), POPE (blue), POPS (red), PI-3P (green), PSM (wine red), and CHOL (orange).

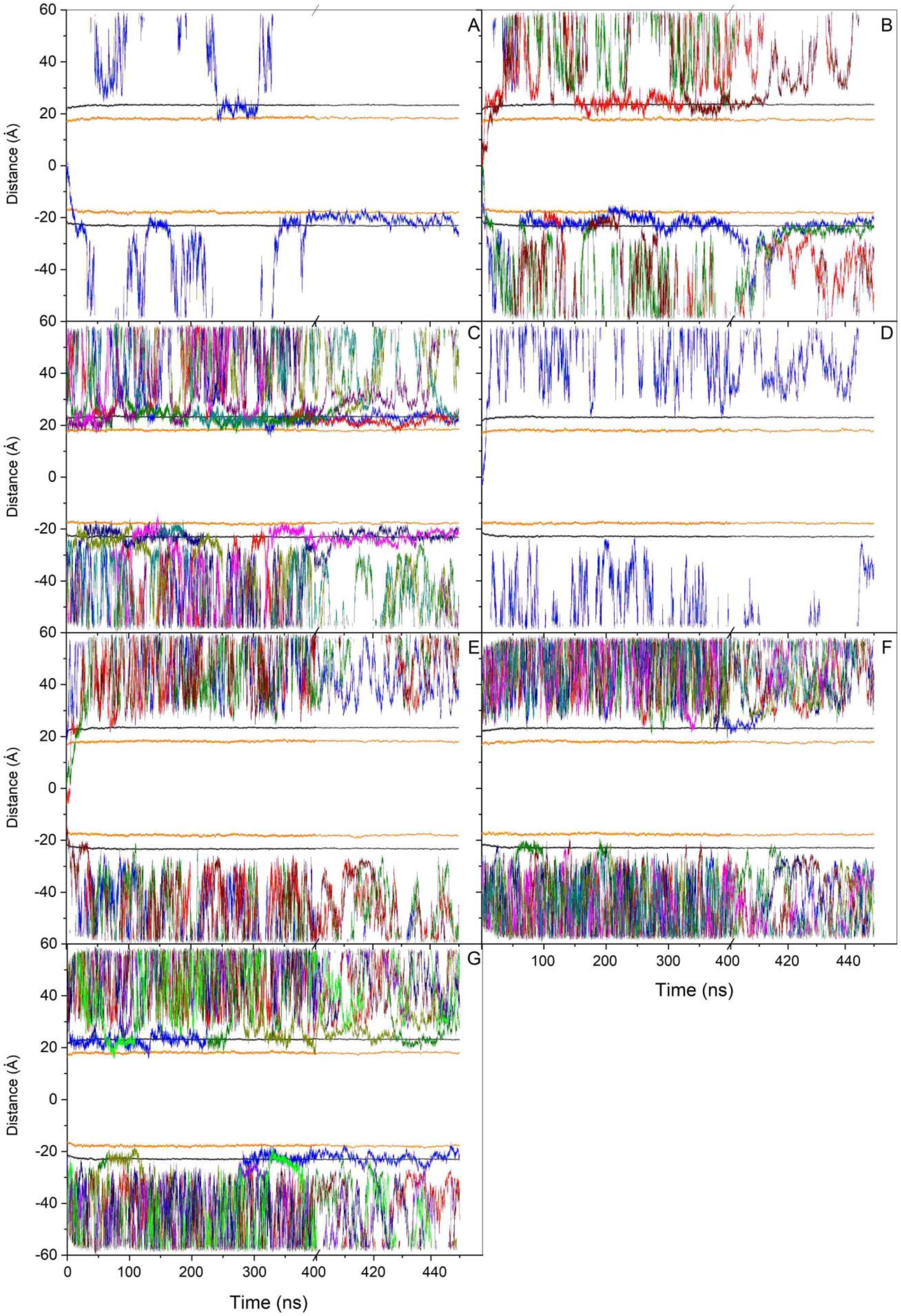

A
B
C
D
E
F
G
Distance (Å)
Time (ns)

Figure S3. Time variation in the whole-molecule ERGO and ERGT z-axis COM distance in relation to (A) system 1, (B) system 2, (C) system 3, (D) system 4, (E) system 5, (F) system 6, and (G) system 7. ERGO and ERGT molecules are represented by different colours. The phosphate atoms of the phospholipids and the oxygen atom of cholesterol are shown in black and orange colours, respectively.

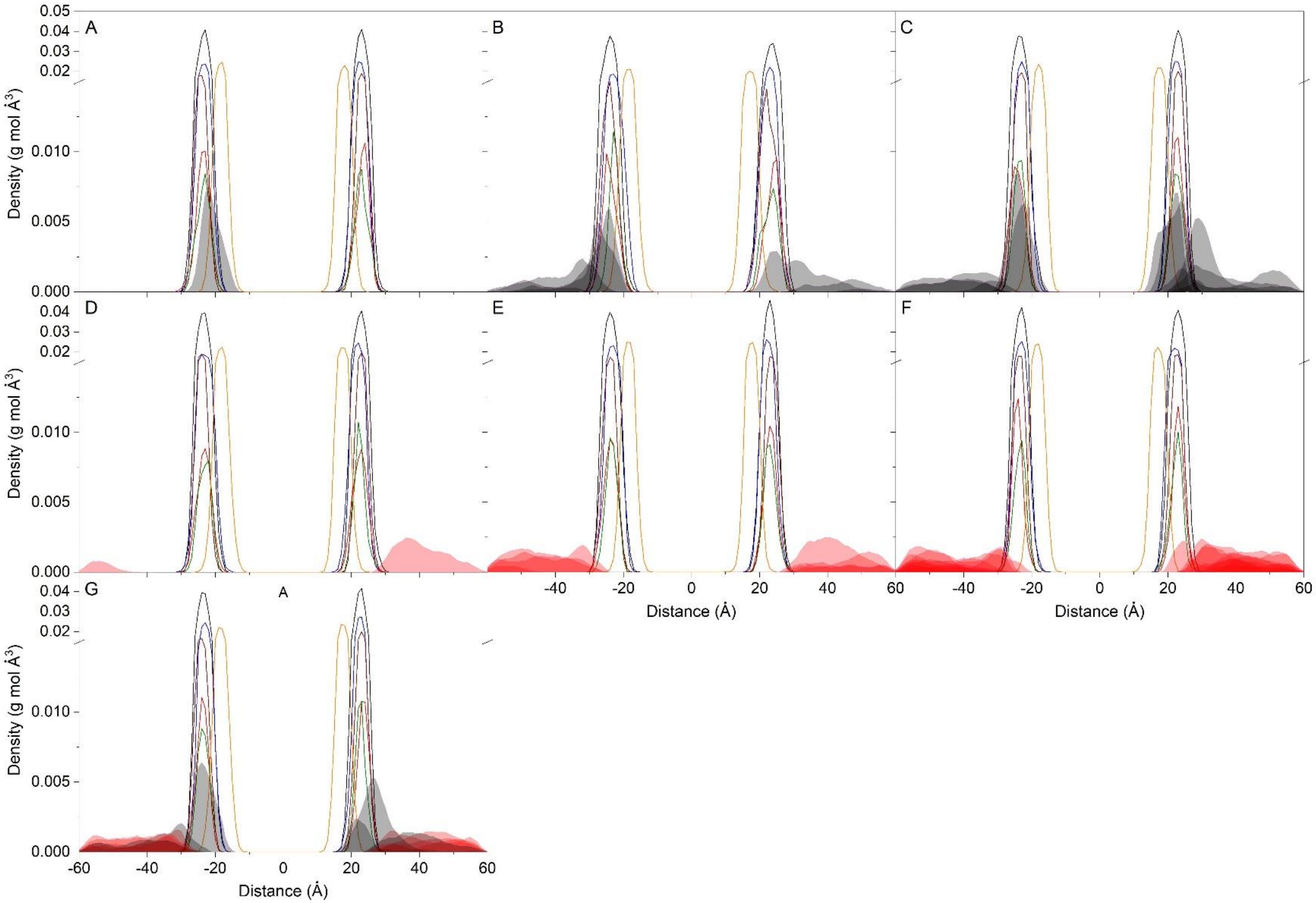

Figure S4. Mass density profiles for the last 30 ns of the MD for (A) system 1, (B) system 2, (C) system 3, (D) system 5, (E) system 6, (F) system 7, and (G) system 7. The phosphate atoms of the phospholipids and the oxygen atoms of CHOL: POPC (-), POPE (-), POPS (-), PI-3P (-), PSM (-), and CHOL (-). The ERGO and ERGT molecules are shown in grey and red filled curves.

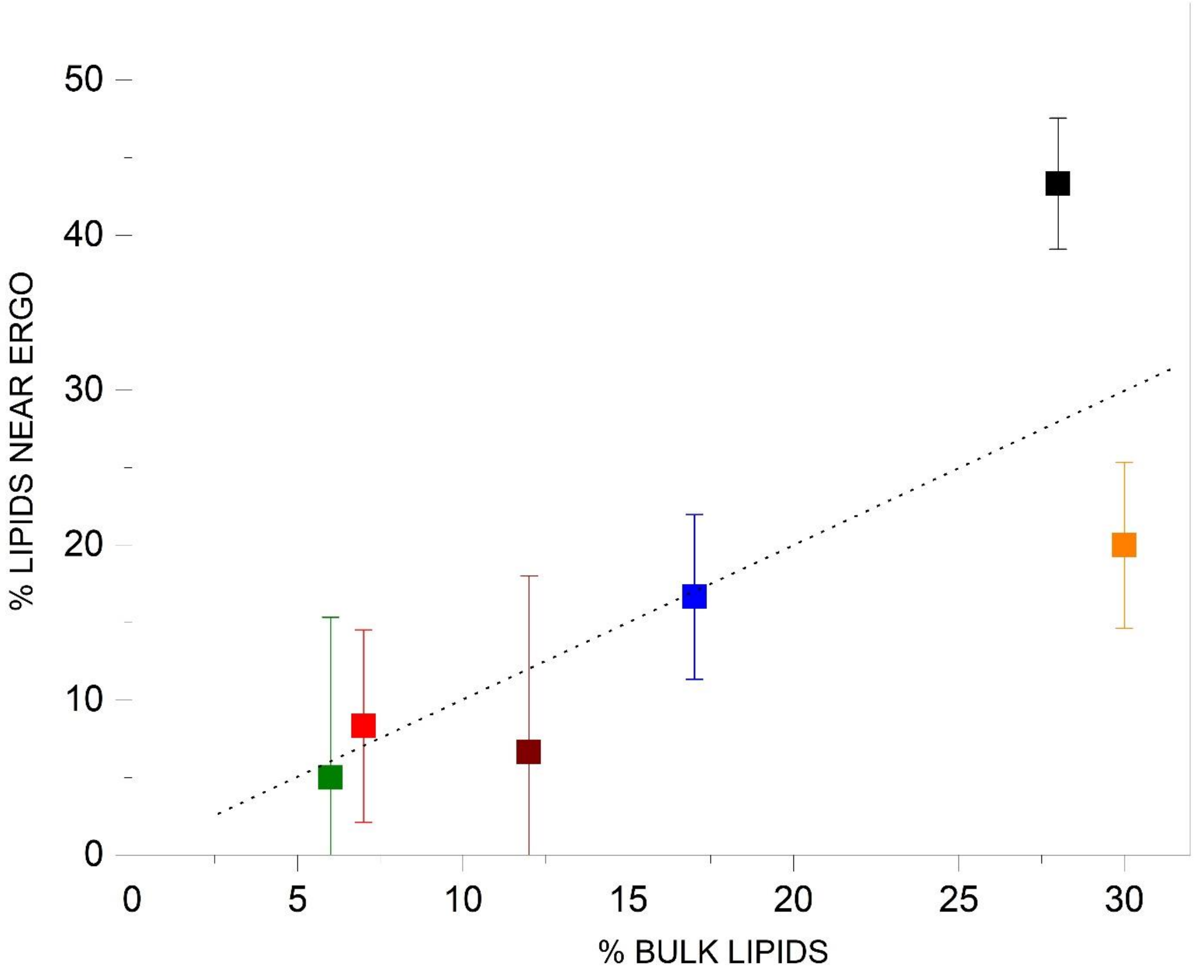

Figure S5. Percentage of the number of lipid molecules in the membrane versus observed number of lipid molecules at a distance of 5 Å from the ERGO molecule in systems 1, 2, 3, and 7: POPC (-■-), POPE (-■-), POPS (-■-), PI-3P (-■-), PSM (-■-), and CHOL (-■-). The dotted line represents identical observed versus expected number of lipid molecules. The analysis was carried out for the last 30 ns of simulation.

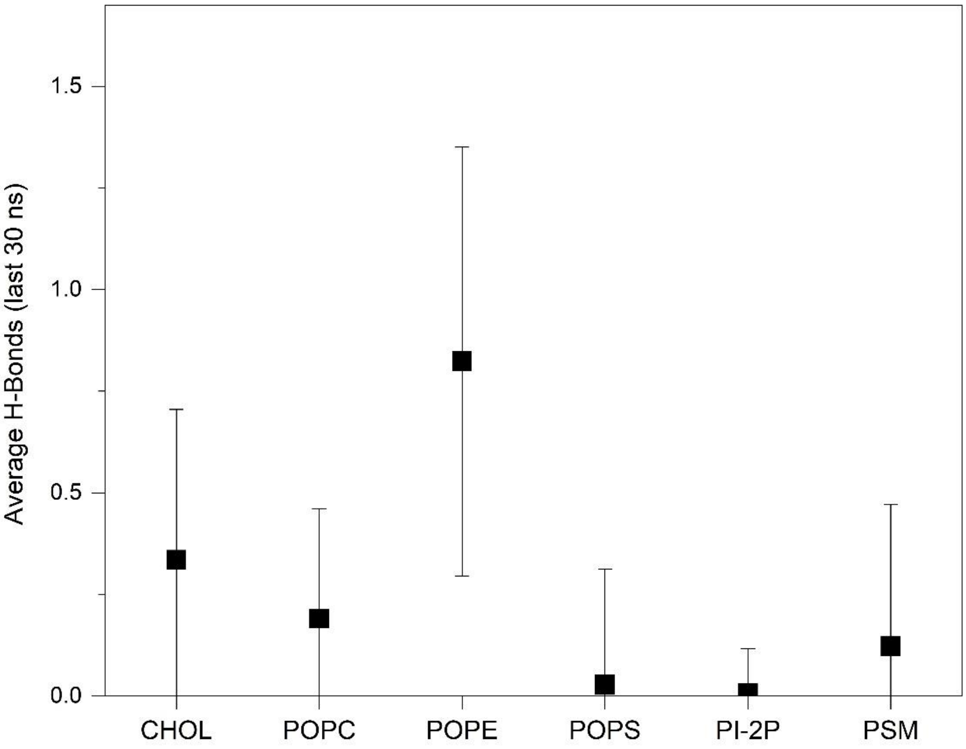

Figure S6. Mean number of hydrogen bonds between membrane lipids and ERGO (eight molecules from systems 1, 2, 3, and 7). The analysis was carried out for the last 30 ns of simulation, and uncertainties represent standard deviation.

Table S1. Average molecular area (Å^2) and membrane thickness (Å) for the last 40 ns of the simulation for all the lipids in the systems studied in this work (mean ± SD).

		AREA (Å^2)						THICKNESS (Å)
	SYSTEM	POPC	POPE	POPS	PI-3P	PSM	CHOL	
**1**	ERGO	52.9 ± 0.8	51.5 ± 1.4	51.6 ± 1.6	49.0 ± 2.8	47.0 ± 1.2	28.2 ± 0.8	46.4 ± 0.2
**2**	ERGO	52.5 ± 0.9	50.7 ± 1.5	51.5 ± 1.7	50.7 ± 1.8	47.4 ± 1.3	27.5 ± 0.7	46.7 ± 0.3
**3**	ERGO	53.6 ± 1.0	52.5 ± 1.4	52.7 ± 2.3	50.9 ± 2.3	46.8 ± 1.4	26.5 ± 0.7	46.3 ± 0.2
**4**	ERGT	53.6 ± 1.0	52.8 ± 1.1	53.3 ± 1.8	51.5 ± 2.3	47.8 ± 1.8	27.0 ± 0.8	45.9 ± 0.3
**5**	ERGT	53.0 ± 0.8	51.8 ± 1.2	52.3 ± 2.2	51.7 ± 2.1	47.1 ± 1.2	26.9 ± 0.6	46.6 ± 0.2
**6**	ERGT	53.3 ± 1.3	51.8 ± 1.6	53.1 ± 2.1	52.3 ± 2.2	49.0 ± 1.2	27.5 ± 0.6	46.0 ± 0.2
**7**	ERGO / ERGT	52.9 ± 1.2	52.6 ± 1.1	53.4 ± 2.0	51.4 ± 2.0	49.3 ± 1.3	26.8 ± 0.9	46.2 ± 0.2
